# Biological Prescience: The Role of Anticipation in Organismal Processes

**DOI:** 10.3389/fphys.2021.672457

**Published:** 2021-12-17

**Authors:** Carrie Deans

**Affiliations:** Entomology Department, University of Minnesota, St. Paul, MN, United States

**Keywords:** prediction, cephalic responses, feed-forward control, allostasis, non-cognitive, microbe, physiological regulation

## Abstract

Anticipation is the act of using information about the past and present to make predictions about future scenarios. As a concept, it is predominantly associated with the psychology of the human mind; however, there is accumulating evidence that diverse taxa without complex neural systems, and even biochemical networks themselves, can respond to perceived future conditions. Although anticipatory processes, such as circadian rhythms, stress priming, and cephalic responses, have been extensively studied over the last three centuries, newer research on anticipatory genetic networks in microbial species shows that anticipatory processes are widespread, evolutionarily old, and not simply reserved for neurological complex organisms. Overall, data suggest that anticipatory responses represent a unique type of biological processes that can be distinguished based on their organizational properties and mechanisms. Unfortunately, an empirically based biologically explicit framework for describing anticipatory processes does not currently exist. This review attempts to fill this void by discussing the existing examples of anticipatory processes in non-cognitive organisms, providing potential criteria for defining anticipatory processes, as well as their putative mechanisms, and drawing attention to the often-overlooked role of anticipation in the evolution of physiological systems. Ultimately, a case is made for incorporating an anticipatory framework into the existing physiological paradigm to advance our understanding of complex biological processes.

## Introduction

The concept of anticipation is deeply rooted in the foundations of science. It lives at the intersection of ideas on time, change, perception, and causality, which encompass some of the oldest philosophical contemplations. As such, it has been addressed in a multitude of different fields, including gnoseology and epistemology (Burgers, 1975; Rosen, 1985a), mathematics (Rosen, 1978; Zadeh, 1978; Dubois, 2003), physics (Whitehead, 1929; Feynman, 1982), economics (King, 1938; Klir, 2002), computer science (Svoboda, 1960; Feynman, 1982; Zadeh, 2001), biology (Libet et al., 1983; Libet, 1985; Rosen, 1985a,b), neurophysiology (Bernstein, 1935, 1945, 1947), and psychology (Hoffmann et al., 2007; Butz, 2008; Bubic et al., 2010). Despite its recent popularity in the computer sciences, particularly relating to information theory, algorithm development, and machine learning, the concept of anticipation is perhaps most widely discussed in the field psychology. This is not surprising given that anticipation is an everyday feature of human thought and one with undeniable value. It is so highly regarded, in fact, that the ability to anticipate with a high degree of accuracy is widely regaled as a supernatural feat by cultures around the world. Although anticipation has been studied in non-human organisms, perhaps most famously by work of Pavlov (1902) on cephalic responses in canines, which he described as “psychic secretions,” the reality is that you would be hard-pressed to find a discussion of it in the broader biological sciences, particularly outside of research on psychology and cognition. Increasing evidence, however, shows that anticipatory processes are not simply reserved for those organisms with brains or complex neural systems. Anticipatory genetic networks have been documented in yeast and bacterial species (Shudo et al., 2003; Tagkopoulos et al., 2008; Mitchell et al., 2009), while other putative anticipatory processes can be found in plants (Karban et al., 2003; Engelberth et al., 2004; Kessler et al., 2006; Falik et al., 2011) and archaea (Whitehead et al., 2009). Even simple biochemical networks can themselves display anticipatory properties (Tagkopoulos et al., 2008; Mitchell et al., 2009; Freddolino and Tavazoie, 2012). These findings suggest that anticipation is not a pinnacle of evolutionary innovation but rather a fundamental type of biological mechanism that likely evolved very early on, and contrary to human superstition, is not at all supernatural but rather entirely natural (Westerhoff et al., 2014; Calvo and Baluška, 2015; Novoplansky, 2016).

If anticipatory processes, i.e., those possessing predictive regulatory structures, are so prevalent, why then, is anticipation such an elusive concept in the biological sciences? Despite many potential reasons for this, it is not from a lack of consideration. The concept of anticipation, as it relates to biology, has indeed been studied by many academics. One of the most thoughtful and prolific writers on the subject is Robert Rosen. His seminal book Anticipatory Systems (Rosen, 1985a) provided a rigorous epistemological and mathematical justification for the existence and importance of anticipatory systems. Rosen asserted that anticipatory systems had been largely overlooked in modern science due to the field’s preoccupation with reductionism and Newtonian physics; a paradigm that only allows present states to be dependent on past or current states, but never future states. Rosen believed that this restriction is empirically and philosophically erroneous and ultimately prevents modern science from ever being able to explain complex biology systems. As a student of relational biology, which would later produce the field of systems biology, he believed that focusing on biological organization was essential, and that anticipatory systems are most visible at the organizational level.

In addition to these philosophical conflicts, a substantial body of work on the theoretical aspects of anticipation can also be found in the literature. Unfortunately, explicit links between this work and empirical biological data are rare. That is not to say that biological responses possessing anticipatory characteristics have gone unidentified. They have simply been described without a clear association to anticipation. For example, evidence of circadian rhythms was first discovered in the early 1700s (Albrecht, 2010; Bhadra et al., 2017), the biphasic dose–response curve in the late 1800s (Calabrese, 2002), and cephalic digestive responses at the turn of the 20th century (Pavlov, 1902; Powley, 1977; Smith, 1995; Power and Schulkin, 2008). These, and many others putative anticipatory responses, have been known for quite some time but have been studied in isolation with little reason to connect them to each other based on a shared anticipatory design. This is perhaps not surprising, as forthcoming examples will show that it is very difficult to identify the organizational properties that define anticipatory processes, particularly when utilizing a reductionist perspective. It requires rigorous experimentation and the integration of detailed ecological, physiological, and molecular information, which is often unavailable. In many cases, anticipatory responses are routinely assumed to be reactive (sequential and unidirectional) based on logical, yet often circumstantial, evidence. For instance, it would seem unlikely to suggest that gastric enzymes were secreted before the ingestion of food, as is seen in cephalic responses, because their function is so clearly tied to a dietary substrate? It would seem illogical to expect, let alone, test for such a phenomenon. Moreover, the propensity to search for such phenomena is made even less likely by the presiding and pervasive assumption that biochemical reactions are always unidirectional and sequential, which is why most of the responses that can now be described as anticipatory have been, up to this point, viewed as biological outliers. Despite this, recent empirical data are improving our understanding of these processes and making their identification easier.

This review unites new and old data on anticipatory processes to highlight the important role that they have played in the evolution of organismal physiology. This synthesis focuses on non-cognitive models to show that anticipatory processes are older and more ubiquitous than previously thought, and lays the groundwork to for identifying the criteria that can be used to distinguish them from reactive processes. In fact, a primary point of this article is that this distinction must be made to advance our understanding of complex biological systems and the physiological mechanisms underlying them. Doing this, however, requires establishing a biologically-explicit anticipatory framework that satisfies three basic criteria. First, conceptual criteria for defining anticipatory processes must be developed. Second, evidence for the existence of these processes must be demonstrated within an evolutionary context. Third, a justification for the incorporation of an anticipatory framework into the current biological paradigm must be given, including an appraisal of how it advances our understanding of complex biological systems. These three criteria closely follow the structure of this article. The first section discusses the causal aspects of anticipation and discusses key considerations at different biological scales. The second section provides examples of different types of anticipatory processes, particularly in non-cognitive organisms. The last section explores the potential mechanisms used in anticipatory responses, discusses the direction of future research, and highlights how an anticipatory framework can advance our understanding of basic biological/physiological principles.

## What is Biological Anticipation?

In his book Anticipatory Systems (1985), Robert Rosen defines an anticipatory system as one whose current state depends upon a future state, and which occurs when a system contains a model of itself that unfolds in faster than real time. Since then, many have made adjustments to Rosen’s definition. Amendments of Dubois and Resconi (1992) and Nadin (2010, 2016) asserted that anticipatory systems can take into account past, current, and future states, as well as possible future states, which highlights the importance of predictive models with no contingencies. Making predictions about future states does not require any information about the future, but instead can arise from predictive models that incorporate information about the past and present to form a probabilistic forecast of future scenarios. Because this predictive process occurs in real time and is in no way contingent on the accuracy of the model, anticipation can clearly be established in the realm of deterministic science. It is important to note that the terms anticipation and prediction will be used synonymously throughout this article. Some of the phenomena discussed may be described by other authors as “predictive,” but there is certainly no need to create unnecessary semantical obstacles in the discussion of like topics. The term “anticipatory,” however, has been specifically selected instead of “predictive” to describe the aforementioned biological processes because its connotation emphasizes a purposiveness that has clear biological and evolutionary relevance. This language also emphasizes the importance of the predicted state over the potentially inconsequential act of making a prediction.

In the context of cognition, anticipation is not controversial, as human beings make predictions on a daily basis. However, the notion that non-cognitive organisms or even biochemical networks can operate in a predictive manner is arguably more provocative. This is especially true given that the language associated with anticipation and prediction is so closely tied to the process of thinking. But what is the process of thinking if not a network of biochemical reactions, subject to the same evolutionary constraints as any other process? Acknowledging the biochemical nature of cognition makes the prospect of more primitive anticipatory responses more acceptable. To truly understand the different facets of anticipation in a biological context, we must start with clear causal principles and identify how these principles operate at different levels of biological organization.

### Causal Considerations

If anticipatory systems are those whose current state is in some way dependent on a future state, then a more biologically explicit definition would describe anticipatory biological processes as those operating in the present to provide an anticipated future function. Here, biological processes are viewed as systems, sometimes anticipatory systems, but it is important to note that living systems are different in many ways from the non-living systems. Most notably, biological processes are subject to natural selection, and this narrows the conditions under which anticipatory biological processes can develop, constrains their designs, and places conditions on the utility of these processes. The implications of these factors will be discussed in more detail below. On a broader scale, however, anticipatory processes can be defined by the role that time and information play in these responses. A fundamental aspect of anticipation is time; thus, any definition of anticipatory systems or processes must contain a temporal element. The definition above makes a causal connection between processes occurring in the present and their dependence on a predicted future state. This is in contrast to processes that occur in the present but are only dependent on events that have already happened. This temporal distinction provides a key division between anticipatory processes and non-anticipatory, or what will be termed reactive, processes.

Information provides another defining factor, as the prediction of a future state is ultimately dependent on it. Information theory describes the relationship between information and uncertainty. The greater the possible states a system is capable of being in, the greater the uncertainty surrounding its state at any given time. Acquiring information about the system or anything that affects it can reduce this uncertainty. In a reactive system that operates based on what has already happened, there is little uncertainty about what is required. However, an anticipatory system operates based on a potential future state that can only be inferred from past and current information, which produces more inherent uncertainty. Why then, would anticipatory processes be advantageous? The answer may have something to do with the fact that uncertainty is an unavoidable feature of life due to the interaction that living things have with their environment. The majority of biological functions are geared toward maintaining growth and reproduction in variable environments, and thus environmental variability represents a considerable amount of fitness-relevant uncertainty. There is, therefore, strong evolutionary pressure to reduce this uncertainty through the acquisition of information, which is evidenced by the existence of diverse sensory structures and signal transduction pathways.

Acquiring information is only one aspect, however, as processes are designed to utilize different types of information in different ways. Some signaling molecules convey more information than others, while some simply contain different types of information. Signal transduction pathways have evolved to respond to these different qualities, and although they operate in real time, some pathways can respond to signals that carry information about forthcoming events. In reactive processes, little uncertainty exists about the utility of the response because there is a direct temporal relationship between the initiating signal and the need for the response. In anticipatory processes, information about future scenarios can be transmitted through temporal correlations between a current signal and future events. When environmental factors change in predictable ways, the detection of a change in one variable can reliably predict a future change in another, reducing uncertainty, but these correlations are still probabilistic even if highly reliable. For more information about information theory and cell signaling see Brennan et al. (2012).

### Ecological Considerations

In an unchanging environment, it is difficult to imagine a use for anticipatory processes. All physiological functions would be carried out by reactively, as organisms would have nothing to anticipate. On Earth, however, environmental change is common across spatiotemporal scales and because of this, living things must be able to sense and react to these changes. Some organisms do this through reactive, sometimes called reflexive, processes that sense a change, and then elicit a response. Organisms in erratic environments will sometimes switch randomly between responses when environmental fluctuations are quick and difficult to detect (Kussell and Leibler, 2005; Mitchell et al., 2009; Ben-Jacob and Schultz, 2010). When environmental patterns are reliable, however, the ability to predict environmental change is more tenable and can be highly beneficial. The ability to anticipate change can be adaptive under adverse environmental conditions, as advanced preparation can increase an organism’s chances of survival, but also under favorable environmental conditions, as it can improve an organism’s competitive edge. Of course, there are potential costs to sensing different types of signals and risks associated with inaccurate signals. A more robust analysis of the costs and benefits to anticipation can be found in Kussell and Leibler (2005), Tagkopoulos et al. (2008), and Mitchell et al. (2009). Conceptually, anticipatory regulation is most favorable when response times are long and environmental uncertainty is high, as the ability to mitigate changes effectively can be limited (Kussell and Leibler, 2005; Freddolino and Tavazoie, 2012).

**Figure 1:**
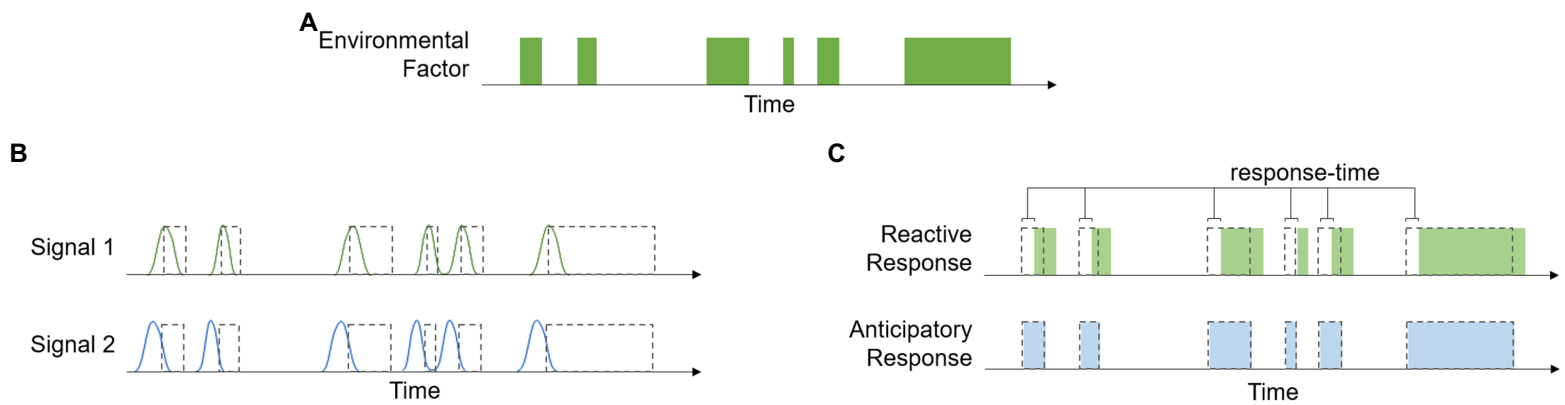
Example showing the time course of a hypothetical environmental factor **(A)**, the time course of two signals that are correlated with this variable **(B)**, and a demonstration of how the temporal relationship between the environmental variable, signal, and response can be used to distinguish between reactive and anticipatory processes **(C)**.

Ecosystems have distinct biogeochemical cycles that often produce reliable environmental patterns. Because of this, many qualitative and quantitative aspects of environmental factors are strongly correlated with each other. When these correlations have a temporal element, they contain information about the time course of environmental events, and biological processes can be designed to take advantage of this information, i.e., anticipatory processes. Figure 1 provides a graphical representation of how these different signals can affect organismal responses. The time course of a theoretical environmental factor is shown in panel (a), and panel (b) shows the time course of Signal 1, a signal with the same temporal pattern as the environmental factor, and Signal 2, which is a signal that is correlated with, but precedes, the environmental factor. Figure 1C shows that because the reactive response is regulated by Signal 1, there is a lag in the response time. This delay is associated with the amount of time it takes to produce the response, such as time related to gene transcription and protein production/transport. The anticipatory response, on the other hand, has no lag in response time because it is regulated by Signal 2, which precedes the change in environment.

Anticipatory processes are most likely to evolve when environmental associations are closely tied to the fitness of the organism, i.e., when they represent a significant constraint on survival or performance. Additionally, the costs of anticipatory, vs. reactive, processes must be less than the fitness benefits obtained through them. In some cases, anticipation may justify the evolution of costly sensory structures, such as brains, but benefits can presumably also be gleaned from simple inexpensive changes in the design of regulatory pathways. As the costs of anticipatory regulation increases, these processes are likely only favorable under high levels of environmental uncertainty (Kussell and Leibler, 2005; Freddolino and Tavazoie, 2012).

A good example of these ecological considerations can be found in work of Tagkopoulos et al. (2008) with *Escherichia coli*. The life cycle of *E. coli* involves the movement of the bacteria from soil, into the mouth of a host, and eventually into the intestines, during which there are subject to predictable fluctuations in environmental temperature and oxygen. Initial ingestion is accompanied by an increase in temperature, and as the bacteria travels to the intestines, it experiences a shift from an oxygen-rich to an oxygen-poor environment. Thus, there is a strong temporal association between the increase in temperature and an impending drop in oxygen availability. These factors have important ecological implications for *E. coli*, as switching from aerobic to anaerobic respiration is an energetic and time-consuming process, also one that if not timed properly, can result in death. This example demonstrates how a temporal correlation between two fitness-relevant factors could favor the evolution of anticipation. Tagkopoulos et al. (2008) did indeed show that *E. coli* transcriptionally regulate metabolic genes in an anticipatory fashion when exposed to increases in temperature. In fact, they showed experimentally that after temperature is increased, genes related to aerobic respiration were significantly repressed while those related to anaerobic respiration were significantly upregulated, even when the current oxygen levels were still high.

### Physiological Considerations

While the ecological considerations outline the utility for anticipation in evolutionary terms, anticipatory processes are ultimately carried out by physiological responses. One of the defining features of life is its opposition to entropy. In a dynamic environment, organisms must maintain order within themselves, a feature epitomized by the concept of homeostasis, which refers to an organism’s internal steady state. It is also often defined as the process by which this internal state and proper physiological function is maintained after perturbations occur. Inherent to the idea of homeostasis is physiological constancy and the existence of static biological optima, or setpoints (Cannon, 1929; Moore-Ede, 1986). Homeostasis, as a process, operates by detecting deviations away from these setpoints, termed error, and eliciting responses to return setpoints back to their original state. Homeostasis can be described as a reactive process that is only initiated after error occurs. However, the observation that many traits in humans and other animals, including blood pressure, insulin secretion, body temperature, etc., vary consistently throughout the day, challenged the notion of static setpoints. Additionally, the existence of physiological trade-offs contradicts the idea that setpoints must be unchanging for homeostasis to be reached. Over time, new terminology was created to account for these dynamic aspects of physiological regulation, including homeorhesis (Waddington, 1958, 1968; Bauman and Currie, 1980; Baumgard et al., 2017), heterostasis (Selye, 1975), homeokinetics (Soodak and Iberall, 1978), rheostasis (Mrosovsky, 1990), but few terms have caught on in popular literature.

The reactive nature of homeostasis was questioned by Moore-Ede (1986), after noticing that the diurnal cycles of many physiological processes, including body temperature, plasma cortisol levels, growth hormone levels, and urinary potassium excretion, began before changes in light cues occurred and/or ahead of the sleep–wake cycles that were assumed to be regulating them (Moore-Ede et al., 1982; Fuller et al., 1985). This led to the conclusion that these traits were regulated by a form of predictive homeostasis, a process that was more efficient because it minimized error. Sterling and Eyer (1988) built off of this idea to produce the concept of allostasis, which places fitness, rather than constancy, as the primary objective of physiological regulation (Sterling, 2004). In contrast to homeostasis, allostasis affirms that fitness is primarily maintained through physiological flexibility because optima are constantly changing across different environments. As a result, the best way to reduce error is to pre-set optima to predicted levels based on an expected physiological response drawn from past experience (Sterling and Eyer, 1988; McEwen, 1998; Schulkin, 2003; Sterling, 2004). Because environmental factors are often temporally correlated, using environmental information to adjust setpoints produces more efficient responses than keeping setpoints at some average level, making an allostatic model more evolutionarily advantageous, and thus more plausible. Although not a commonly discussed concept, the allostatic perspective has in fact improved our understanding of the regulation of several traits, in addition to providing a strong foundation for anticipatory regulation (Tagkopoulos et al., 2008; Mitchell et al., 2009; Perkins and Swain, 2009; Freddolino and Tavazoie, 2012).

Regulatory mechanisms are the most physiologically relevant aspects of anticipatory processes and can be delineated by stimulus–response (S-R) relationships. It is important to note that the accurate detection of stimulus–response relationships is imperative to discerning between reactive and anticipatory processes, but these relationships are not always easy to detect, especially in complex regulatory networks. Mitchell et al. (2009) provides a nice framework for conceptualizing the regulatory differences between reactive and anticipatory processes by distinguishing between four types of regulatory strategies: direct regulation, stochastic switching, symmetrical anticipatory regulation, and asymmetrical anticipatory regulation (AAR). Direct regulation occurs when one stimulus activates one response (S1 > R1 and S2 > R2) and is most similar to generalized reactive processes. In stochastic switching, one stimulus activates one of two responses randomly (S1 > R1 or R2). In symmetrical anticipatory regulation (SAR), two stimuli elicit their own responses and the response of each other (S1 > R1 or R2 and S2 > R2 or R1). In AAR, one stimulus activates its own response and the response of another stimulus, while the other stimulus only activates its own response (S1 > R1 or R2 and S2 > Rs).

**Figure 2:**
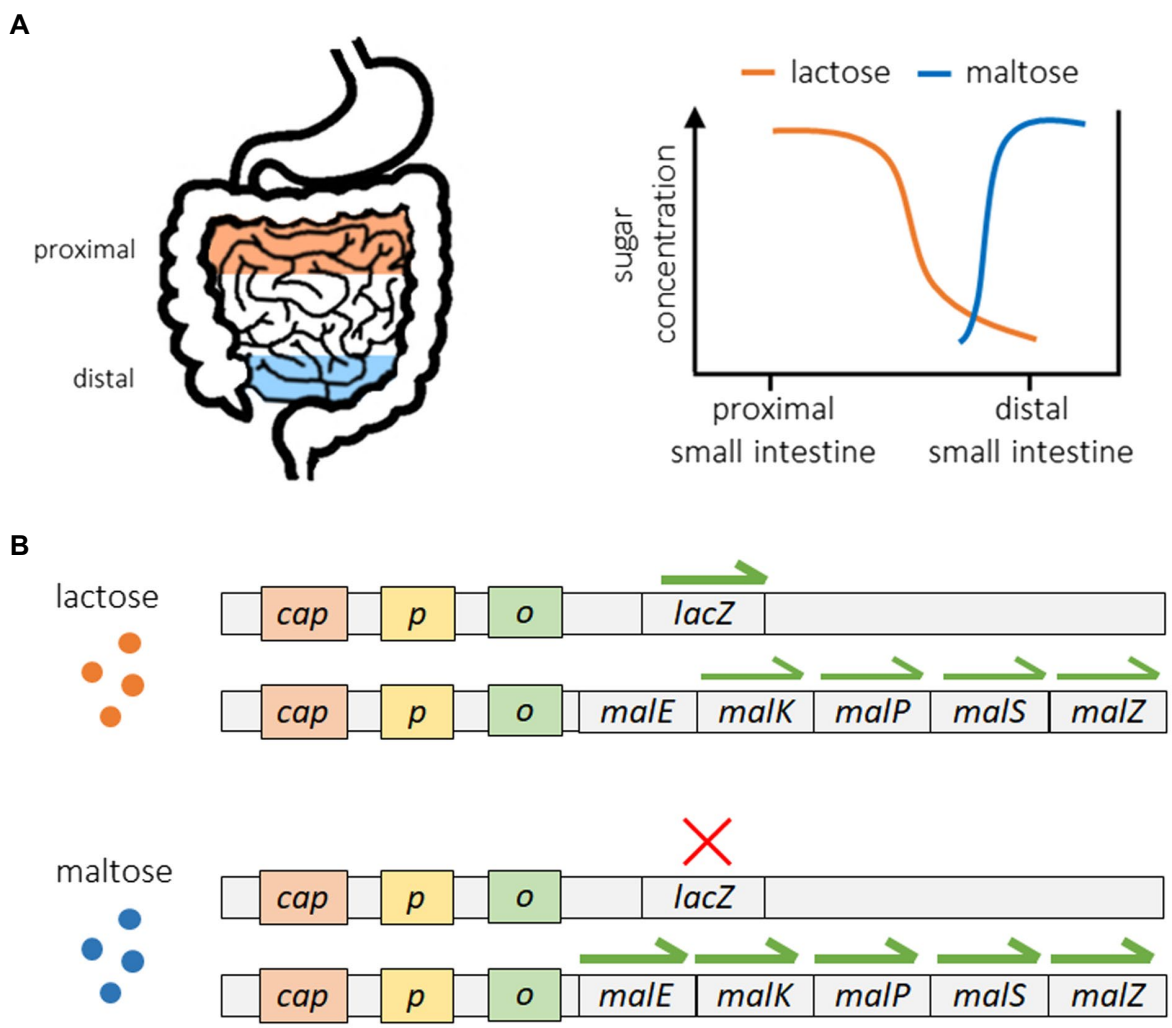
Anticipatory regulation of sugar metabolism in *Escherichia coli*. The movement of *E. coli* from the proximal end of the small intestine toward the distal end is associated with a predictable sequence of exposure, first to lactose followed by maltose **(A)**. Expression of the lac operon occurs in a reactive manner once lactose is present, while the expression of specific maltase genes exhibits anticipatory regulation. Maltase genes are upregulated in the presence of maltose but also significantly expressed upon exposure to lactose in the absence of maltose **(B)**. This regulatory structure induces maltase production in anticipation of future maltose availability and is associated with increased fitness. The data shown here are from Mitchell et al. (2009). Only the expression specific promoters were measured (data for lacY and lacA and only five of the 10 maltase gene promoters are shown).

A good example of these strategies can be seen in the regulation of sugar metabolism in *E. coli* (Mitchell et al., 2009; Freddolino and Tavazoie, 2012). *Escherichia coli* can utilize a range of different sugars for energy acquisition, and in the gut of their hosts they encounter different sugars in a specific order. As shown in Figure 2A, *E. coli* bacteria encounter lactose at the proximal end of the small intestine, while maltose is not encountered until reaching the distal end of the small intestine (Savageau, 1998; Mitchell et al., 2009). Because of this, exposure to lactose always precedes exposure to maltose. Figure 2B shows how the lac operon, which controls the expression of lactase enzymes, operates in a reactive manner. The expression of lactase genes only occurs in the presence of lactose, which serves as a direct stimulus. The mal operon, which controls the production of maltase enzymes, operates in an anticipatory manner, as the expression of maltase is stimulated by the presence of maltose (direct stimulus) but also lactose, which serves as an indirect stimulus. Because of this, maltase expression begins when bacteria encounter lactose in the proximal small intestine but before they are ever exposed to maltose. This way, maltase is already present when maltose becomes available, reducing the response time associated with maltase production and increasing the efficiency of maltose catabolism. Although Mitchell et al. (2009) did not directly measure the response time of maltase production under different sugar scenarios, they did show that pre-exposure to lactose significantly increased the fitness of *E. coli* subsequently reared on maltose. Some other examples of putative anticipatory regulation include the cross-protection of yeast to oxidative stress when pre-exposed to heat shock and/or ethanol shock (Mitchell et al., 2009; Dhar et al., 2013), the high-risk foraging behaviors of the slime mold Physarum polycephalum on low quality food sources (Latty and Beekman, 2010), and the upregulation of motility and metabolic genes for preferred carbon sources in *E. coli* reared on lower quality carbon sources (Liu et al., 2005; Zhao et al., 2007).

These generalized regulatory strategies show how differences in S-R relationships can produce reactive and anticipatory processes. Anticipatory processes, however, can also stem from alterations to the quantitative features of S-R relationships, such as stimulus thresholds and response dynamics. For instance, hormetic responses are characterized by a disproportionate response to low levels of stress, where low doses of stress cause an overcompensated response, hypothesized to better prepare the organisms for ensuing higher doses (see section on Hormesis). Priming, also called preconditioning, is another example of a dose-dependent response. Primed responses occur when organisms are exposed to low-moderate amounts of stress that initiate a partial response that is only completed upon exposure to higher levels of stress (see section on Priming). The primed state occurs in anticipation of higher levels of future stress, much in the same way that the overcompensated response in hormesis does. In these examples, a stimulus can produce a reactive or anticipatory response depending on the concentration of the stimulus.

### Biochemical Considerations

All biological processes are fundamentally mediated by biochemical reactions. As such, defining anticipatory processes in biochemical terms is essential for demonstrating their fundamental role in biology. This is quite difficult to do, however, because our understanding of anticipatory mechanisms is still quite limited. Despite this, considerations at broader biological scales can be used to shape our understanding of biochemical criteria, even if only hypothetically. For instance, the causal aspects of anticipation highlight the importance of timing, while physiological considerations provide us with a S-R structure to build off. These components and the constraints they entail allow for the development of hypotheses about the more ambiguous biochemical characteristics.

**Figure 3:**
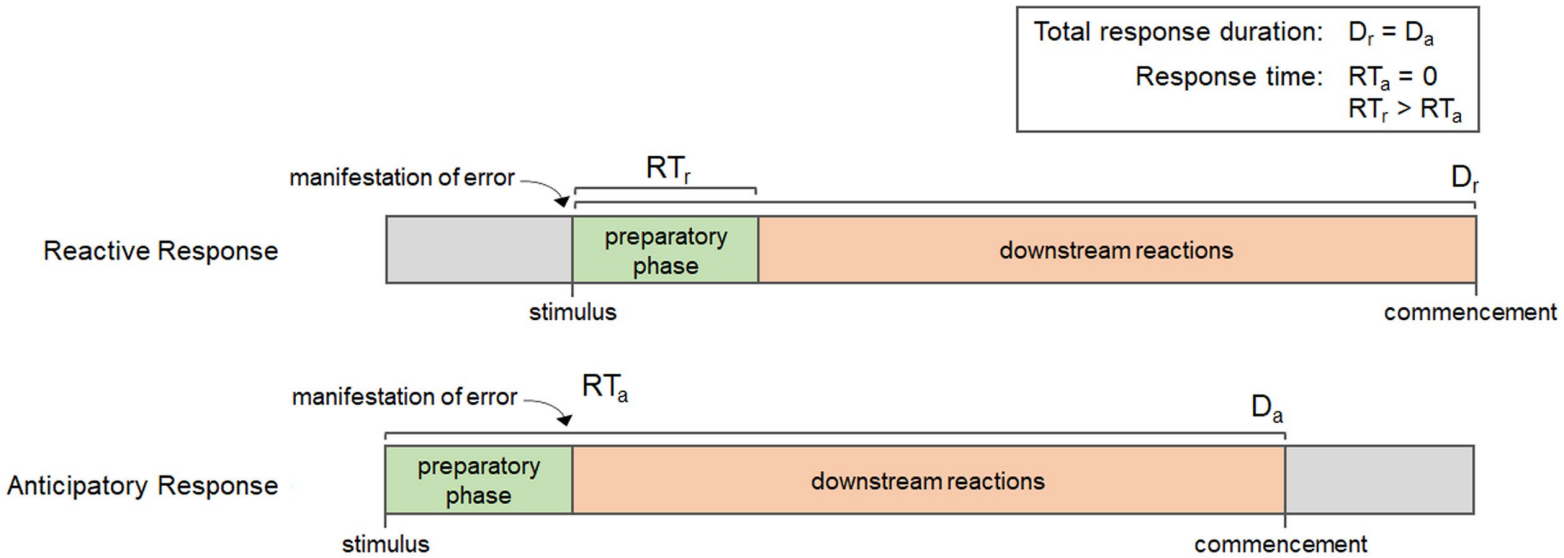
Diagram showing the key events in a reactive response and a generalized anticipatory response, including the manifestation of error, the initiation of the preparatory phase, downstream reactions, and commencement. Notice that although the duration of each response (D_r_ and D_a_) is the same, the anticipatory response has a shorter response time (RT_a_) than the reaction response (RT_r_) due to the initiation of the preparatory phase occurring before the manifestation of error.

Every biological process has a beginning, a middle, and an end, and the timing of the reactions involved at each stage can have important impacts on the efficiency of response. Many physiological responses are only induced when needed, thus, they are initiated once homeostatic error occurs. Once error is detected, there is a preparatory phase where the required components (enzymes, substrates, and/or co-factors) must be produced, transported, and/or concentrated so that downstream reactions can take place. In fact, four important events can be demarcated in the time course of biological responses: the manifestation of error, the initiation of the preparatory phase, the initiation of downstream reactions, and the commencement of the response. The organization of these events affect the overall duration of the response, which is the time between the initiation of the preparatory phase and commencement, as well as the response time, which is the period of time between the manifestation of error and the initiation of downstream reactions; or when error mitigation actually begins. Figure 3 shows the time course for each of these events in a reactive response and a generalized anticipatory response. It is important to note that the duration of the preparatory phase can vary depending on the response, but regardless of whether it is long or short, it results in a delay in the downstream reactions and an increase in response time. Anticipatory processes, however, are coordinated such that all or part of the preparatory phase occurs before the manifestation of error, thereby reducing response times. This is evident in Figure 3, which shows how the early initiation of the preparatory phase reduces the response time for anticipatory processes relative to reactive processes. Using these events, we can then describe anticipatory processes in biochemical terms, as those processes that reduce response times by initiating the preparatory phase before the manifestation of error. These characteristics can be achieved in different ways; either through qualitative or quantitative alterations to their mechanisms.

For instance, processes controlled by AAR represent good examples of qualitative alterations and are useful for developing hypotheses biochemical components. In AAR, there are two types of S-R relationships; two direct relationships (S1 > R1 and S2 > R2) and one indirect relationship (S1 > R2). The differences between direct and indirect stimuli can help produce useful hypotheses regarding the biochemical differences between reactive and anticipatory processes. Direct S-R relationship can be viewed as primary, while indirect stimuli are ancillary. A direct stimulus indicates that a physiological response is needed and elicits the response in real time. To do this, a temporal relationship must exist between the presence of the stimulus and the manifestation of error, which suggests that direct stimuli are likely to be compounds or by-products of the process creating error, such as toxins or antigens. An indirect stimulus, on the other hand, need not have an intimate biochemical relationship with the process creating error or to the direct stimulus. It must simply be temporally correlated with the creation of error and/or the direct stimulus. This suggests that there are far fewer constraints on the chemical properties of indirect stimuli and their relationship to the downstream reactions they elicit.

While some anticipatory processes may utilize different stimuli to change the time course of responses, other processes can simply alter the modulation of the response itself. Priming, which is discussed in more detail in the next section, is a good example of this. Sometimes called sensitization or preconditioning, priming occurs upon re-exposure to a prior stress. From a biochemical perspective, priming shortens response times by initiating and carrying out part of the preparatory phase upon exposure to low levels of stress so that the response is primed and downstream reactions can occur more quickly upon re-exposure. While sensitization mediated by neurological responses is well understood as a form of neural plasticity, the mechanisms underlying other types of priming in non-cognitive species are more ambiguous. In plants, however, priming appears to be mediated by the accumulation of dormant signaling proteins, the storage of metabolite conjugates, and/or changes to epigenetic marks, such as gene methylation and histone modifications. For instance, in Arabidopsis, exposure to both salicylic acid analogues and bacterial infection leads to the accumulation of inactive mitogen-activated protein kinases, specifically MPK3 and MPK6 (Beckers et al., 2009; Conrath, 2011), suggesting that they allow the plant to amplify stress signals faster upon subsequent exposure. The accumulation and storage of other compounds, such as reactive oxygen species (ROS) and metabolite conjugates in vacuoles have also been documented in primed plants (Pastor et al., 2013). Gene methylation has been observed in the priming of barley to powdery mildew exposure (Kraska and Schönbeck, 1993; Conrath, 2011), and histone modifications have been tied to increased expression of defense-related genes in primed responses due to the faster recruitment of transcriptions factors, co-activators, effector protein, and general transcriptional machinery (Alvarez-Venegas et al., 2007; March-Díaz et al., 2008; van den Burg and Takken, 2009; Jaskiewicz et al., 2011). In either case, the same stimulus triggers the response but the reduction in response time is achieved by changes in the modulation of the response after it has been initiated.

### Toward and Integrative Definition of Anticipatory Processes

**Figure 4:**
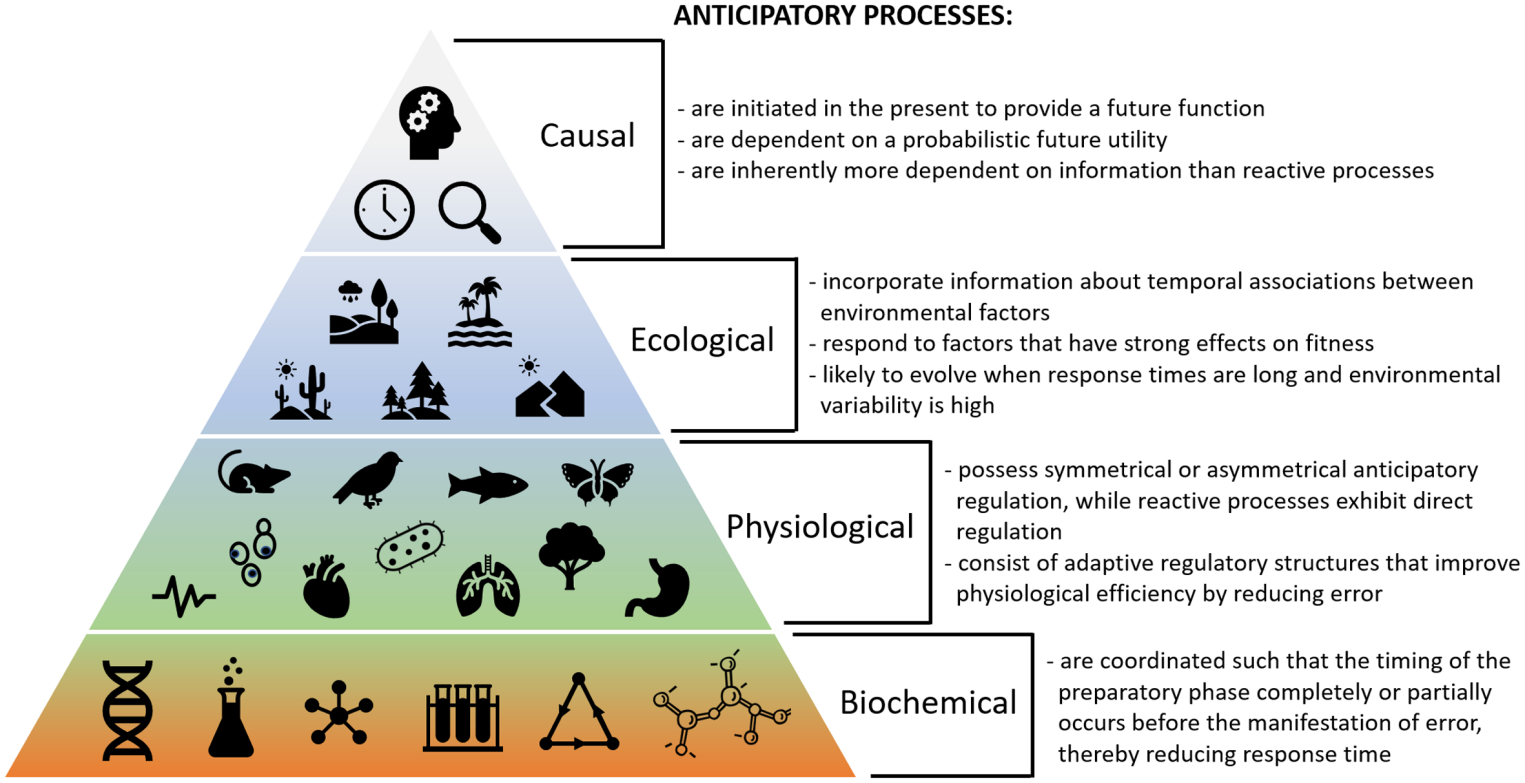
Summary of key criteria for biological anticipation at different levels of organization, including causal, ecological, physiological, and biochemical considerations.

Figure 4 shows potential criteria defining biological anticipation at different scales. These considerations can be used to help identify potential anticipatory processes based on different ecological, physiological, and biochemical criteria. Because reactive processes utilize simpler mechanisms that likely predate anticipatory processes, we can use them as comparators to further understand the characteristics that make a process anticipatory. Moreover, examining processes that exist in both reactive and anticipatory forms can be useful.

If we consider the examples discussed above, we can see how considerations at different scales connect to provide an evolutionary justification for anticipation that is consistent with physiological and biochemical mechanisms. Using AAR as an example, we can imagine a scenario where a physiological response, denoted by a specific S-R relationship, evolves to be regulated by a new stimulus that is correlated with but predates a fitness-relevant environmental variable. This alteration could then cause the preparatory phase of the response to be initiated before error occurs. This generalized scenario combines the criteria for anticipation at all levels of biological organization into a cogent explanation. The next section discusses real biological phenomena that meet some or all of these criteria.

## Examples of Anticipatory Processes

Now that some relevant aspects of anticipation have been discussed at the causal, ecological, physiological, and biochemical levels, we can explore some examples. The following section outlines some key categories of anticipatory processes and provides specific examples, with a bias towards non-cognitive models. It should be noted that this is not an exhaustive list, and the categories may not necessarily be mutually exclusive, i.e., some examples may fall into more than one category. Despite this, the goal of this section is to show how different biological phenomena, often studied using different model systems, in disparate fields, can be united under an anticipatory framework. Many of the examples discussed in this section, while well-established, have proved difficult to explain by reactive mechanisms. Because of this, many have been viewed, for at least a period of time, as biological outliers. They are potentially explicable, however, by relating their characteristics to the anticipatory criteria described above.

### Circadian Rhythms

**Figure 5:**
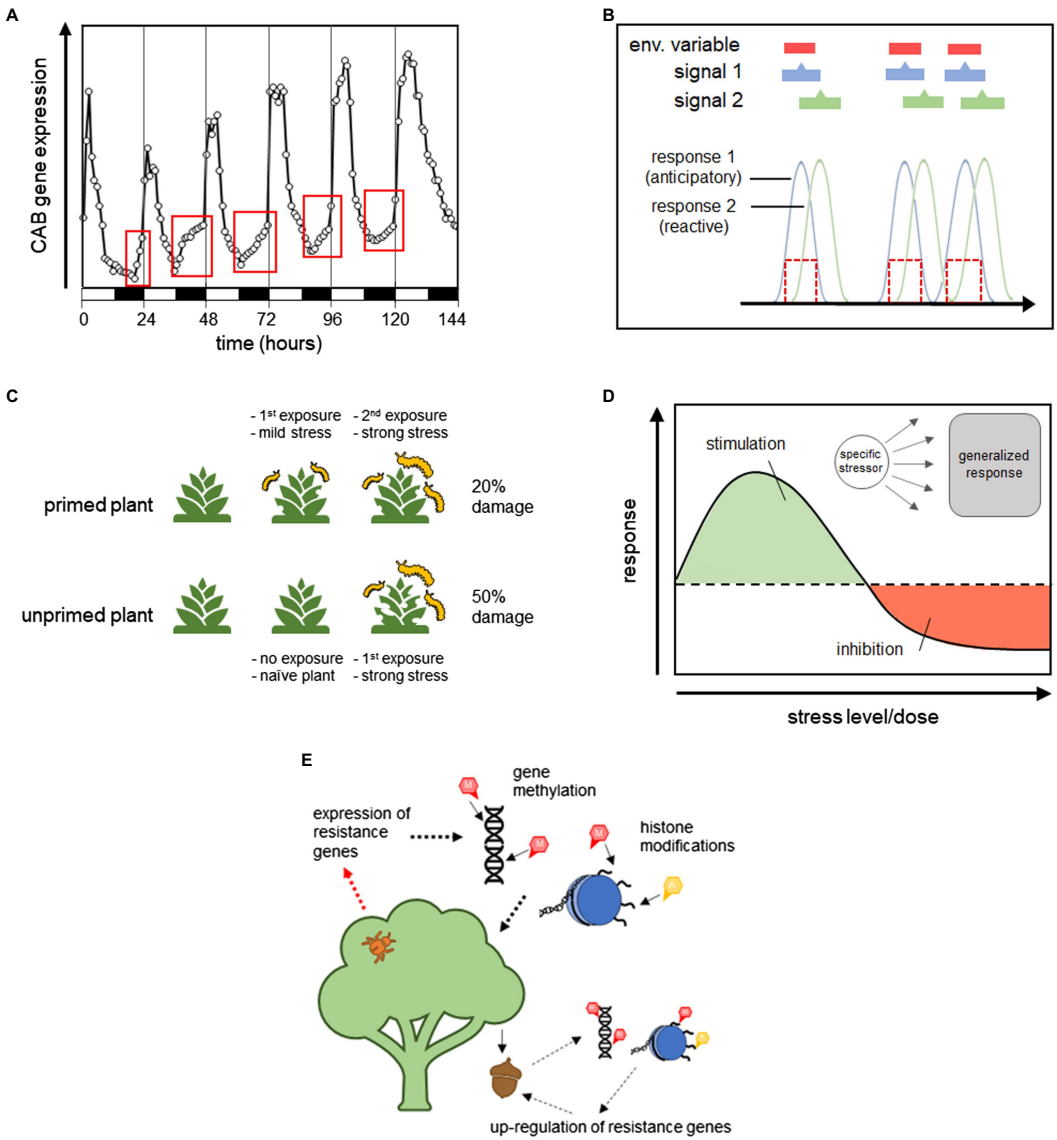
Graphical representation of the different types of anticipatory processes. **(A)** Shows how circadian rhythms can regulate the timing of physiological processes in an anticipatory manner. This figure is adapted from Millar and Kay (1996) and shows the upregulation of chlorophyll *a*/*b*-binding protein (CAB) expression in Arabidopsis seedlings before the morning increase in light availability. **(B)** Shows asymmetrical anticipatory regulation (AAR), which occurs when a response is regulated by a stimulus that is correlated with but predates an environmental variable, allowing the response to be carried out in anticipation of the environmental change. **(C)** Shows priming in a plant-insect model system, where previously attacked, or primed, plants are more resistant than naïve plants to future attack. **(D)** Shows the J-response curve characteristic in hormesis, where an over-response to a specific stressor can provide beneficial protection from other more general stressors. **(E)** Demonstrates how parental expressional states can be passed on to offspring to increase fitness in predicted future conditions *via* transgenerational effects, shown here by heritable gene methylation and histone modifications.

Circadian and circannual rhythms are arguably one of the oldest mechanisms of anticipation. Found in both prokaryotes and eukaryotes, these rhythms are controlled by an internal biological clock that is calibrated to the diurnal (day-night) and annual cycles of the Earth. Regulating physiological processes by an internal clock is more efficient than responding to individual environmental cues because so many environmental factors correlate with diurnal and annual cycles. This not only streamlines regulatory processes and reduces the need for larger more costly sensory networks, but, as argued by Moore-Ede (1986), it also allows for the anticipation of physiological needs. Possessing an internal clock that is calibrated to stable environmental periodicities allows organisms to schedule metabolic functions in the most efficient way, as shown in Figure 5A with the example of photosynthetic compounds in plant increasing in anticipation of daylight. In humans, plasma cortisol and core body temperature upcycle in the morning before any light stimulus is present (Moore-Ede, 1986). The diurnal regulation of insulin (Hara and Saito, 1980) digestive enzymes (Stevenson et al., 1975; Saito et al., 1976a,b), and glucose transport (Stevenson and Fierstein, 1976) in rats have also been shown to be anticipatory, as well as circadian-controlled food seeking behavior in rats and squirrel monkeys (Boulos et al., 1980; Moore-Ede, 1986; Mistlberger, 1994; Stephan, 2001; Davidson et al., 2003). Circadian-based mechanisms temporally coordinate metabolic activities to reduce the lag times associated with reactive responses and thus, meet the criteria for anticipatory processes. This strategy ensures that enzymes, proteins, nutrients, etc., are available at the instant they are needed, maximizing physiological efficiency.

Circadian rhythms are well documented in mammals, insects, fungi, and plants (Millar and Kay, 1996; Harmer et al., 2001; Bhadra et al., 2017), as well as prokaryotes (Johnson et al., 1996; Whitehead et al., 2009; Cohen and Golden, 2015; Nohales and Kay, 2016) and unicellular eukaryotes (Johnson and Kondo, 2010; Mittag and Wagner, 2003; O’Neill et al., 2010), suggesting that they evolved early on and also multiple times in different lineages (Johnson and Kondo, 2010). Traits exhibiting circadian rhythmicity vary widely across taxa and of course not all circadian-controlled traits demonstrate an anticipatory function. Despite this, the basic components of circadian processes are surprisingly similar, consisting of an endogenous central oscillator, input pathways that entrain the oscillator, and output pathways that manifest periodicity in specific traits. Some organisms, such as mammals, possess multiple clocks, including the master pacemaker in the suprachiasmatic nucleus (SCN) and peripheral clocks in other tissues. Despite differences in specific components, all circadian systems rely on delayed negative feedback loops at the transcriptional level (Dunlap, 1999; Harmer et al., 2001), as well as post-transcriptional regulation, to produce the 24-h oscillatory phase. While many clock components exhibit homology across taxa, primary differences in circadian mechanisms relate to variations in specific regulatory factors and complexity, which is more thoroughly reviewed by Harmer et al. (2001) and Bhadra et al. (2017). While the details of entrainment are well-understood for many taxa, our understanding of the circadian regulation of specific traits and its evolutionary history is limited. Elucidating the mechanistic relationship between the central oscillator and the regulation of specific physiological pathways in non-cognitive organisms will be essential for further understanding the evolution of circadian systems and this type of biological anticipation in general.

### Asymmetrical Anticipatory Regulation

As previously discussed, when environmental factors show reliable temporal patterns the presence of one factor can be used to anticipate the future presence of another, much like associative learning. Regulatory pathways across diverse taxa have evolved to take advantage of these correlations through anticipatory processes (Tagkopoulos et al., 2008; Mitchell et al., 2009; Freddolino and Tavazoie, 2012). The regulation of these processes can be altered through changes in signaling, transcription, translation, and post-translational pathways. AAR, as defined by Mitchell et al. (2009), occurs when a response is triggered by an environmental signal that is correlated to and precedes the original stimulus. In this way, the response can be initiated earlier, better preparing the organism for future conditions, as demonstrated in Figure 5B.

Identifying cases of AAR can be incredibly difficult, as it requires having a detailed understanding of an organism’s ecology and the regulatory mechanisms for specific processes. The outcomes of regulatory networks are easy to measure but the mechanisms that produces them are much more difficult to document. Uncharacterized processes are often assumed to possess reactive S-R relationships by default, thereby reducing the discovery of AAR. However, unusual correlations in transcriptional patterns or unintuitive phenotypes can be good indicators that AAR is at play, particularly if the relationship can be explained ecologically. For example, Tagkopoulos et al. (2008) found that *E. coli* genes associated with anaerobic respiration were upregulated when populations were subjected to a spike in temperature. This relationship makes little physiological sense, particularly considering that anaerobic genes increased while oxygen levels were still high; but, when you consider that the ecology of *E. coli* necessitates a move from the mouth of the host, a high-temperature environment, to the anoxic intestines of the host, a functional explanation emerges. The spike in environmental temperature reliably indicates an ensuing anoxic environment, so using a thermal signal to initiate the production of anaerobic cellular machinery better prepares the bacteria for their future environment. Tagkopoulos et al. (2008) also showed that the relationship between temperature and anoxia could be decoupled by rearing bacteria under the opposite conditions (where temperature and oxygen decrease) for less than 100 generations, further substantiating that the transcriptional relationship was an evolved response to an environmental pattern rather than biochemical constraints. Mitchell et al. (2009) reported similar cases of AAR in the sugar metabolism of *E. coli* (Figure 2) and in the stress response of *Saccharomyces cerevisiae* during the wine-making process. Other putative examples of AAR have also been reported in *Vibrio cholerae* (Dunlap, 1999; Schild et al., 2007), *Candida albicans* (Rodaki et al., 2009; Mitchell and Pilpel, 2011), *Mycobacterium tuberculosis* (Mitchell and Pilpel, 2011), and other stress responses in *S. cerevisiae* (Berry and Gasch, 2008; Eoh et al., 2017).

Processes that fit the description of AAR can be found in the scientific literature as early as the turn of the century, such as work of Pavlov (1902) on cephalic responses (Smith, 2000; Power and Schulkin, 2008), but these more recent microbial studies provide new data on AAR in non-cognitive species. Another example of AAR from a non-cognitive organism, is plant eavesdropping. Plants have the ability to pick up on above- and below-ground cues from neighboring plants about potential threats, such as herbivory or drought, and use them to induce defensive responses before exposure to stress occurs (Karban et al., 2003; Engelberth et al., 2004; Kessler et al., 2006; Falik et al., 2011). The majority of work on plant eavesdropping has been done on herbivore-induced plant volatiles, but signals emitted by roots and microbes in the soil have also been studied (Falik et al., 2011; Morrell and Kessler, 2014; Blande, 2017; Rebolleda-Gómez and Wood, 2019). When plants are damaged, the composition of their volatiles change and can serve as reliable signals of impending pest presence. These cues can either elicit direct induction of defenses or prime defense pathways (Morrell and Kessler, 2014). Eavesdropping allows plants to respond to the volatile cues that precede stress rather than the physical (plant damage) and/or chemical (insect saliva) cues that are only present after damage has occurred.

### Priming

Priming, which was also discussed earlier, refers to the enhanced response to a stressful stimulus that has been previously encountered. Initial exposure to a stressful agent can elicit a direct response but can also prime future responses to the same or different stimuli. Priming causes a response to be partially initiated, paused, and only completely carried out after a secondary exposure. In this way, stress responses can be used therapeutically and carried out faster and with greater efficacy. Figure 5C shows a hypothetical example of priming for resistance to herbivory in plants, where a plant that has experienced herbivory in the past is better able to respond to subsequent pest pressure than plants that have never been exposed. Priming can also be seen as a fail-safe to ensure that costly processes are employed economically and only carried out when absolutely necessary. Primed traits can persist over the short-term or long-term, mediated by different mechanisms. Given that priming is implemented as a response to future scenarios and it increases biological efficiency by reducing response time, it qualifies as an anticipatory process.

The most cohesive body of work on priming in a non-cognitive organism comes from the study of induced plant defenses. In plants, exposure to pathogenic and apathogenic bacteria (Jung et al., 2009; Balmer et al., 2015), fungi (Molitor et al., 2011; Gamir et al., 2012; Martínez-Medina et al., 2013), oomycetes (Massoud et al., 2012; Lim et al., 2013), nematodes (Hao et al., 2012; Vos et al., 2013), arthropod damage (Smith and Clement, 2012; Vos et al., 2013), egg oviposition (Kim and Felton, 2013), abiotic stressors (Tuteja and Sopory, 2008; Suzuki et al., 2012; Virlouvet et al., 2018), and synthetic effectors (Conrath, 2011; Bektas and Eulgem, 2015; Conrath et al., 2015) can prime plant defense pathways, conferring increased resistance to a wide range of stressors compared to unprimed plants. While constitutive and direct induction of plant defenses can be costly, primed plants exhibit full protection with fewer fitness costs due to flexibility that priming offers (van Hulten et al., 2006; Wang et al., 2015).

The pathways and processes responsible for priming in plants are diverse, as are the post-challenge responses of primed states. Short-term mechanisms of priming include the accumulation of signaling proteins or metabolite conjugates, while long-term priming results from changes to epigenetic markers. Plant defense metabolites, such as ROS and pathogenesis-related proteins (PRs), inactive conjugated plant signaling hormones [ethylene (Et), jasmonic acid (JA), abscisic acid (ABA), salicylic acid (SA)], microbe-associated molecular pattern (MAMPs) receptor abundance, and mRNA of other signaling components [mitogen-activated protein kinases (MAPKs, MEKs, and MEKKs)] have all been shown to accumulate in the tissue of primed plants, allowing for faster activation and reduced response times upon subsequent exposure (Beckers et al., 2009; Conrath, 2011; Pastor et al., 2013). Additionally, changes in chromatin structure, *via* histone modifications and DNA methylation, have been correlated with the increased expression of defense genes due to enhanced accessibility and recruitment of transcription co-factors and general transcription machinery (RNAPII, TFIID; Alvarez-Venegas et al., 2007; March-Díaz et al., 2008; van den Burg and Takken, 2009; Conrath, 2011; Jaskiewicz et al., 2011). Specifically, the methylation and acetylation of lysine residues on histones H3 and H4 (H3K4me3, H3K4me2, H3K9ac, H4K5ac, H4K8ac, and H4K12ac) and the replacement of H2A with H2A.Z have been tied to increased expression of defense genes in *Arabidopsis* (Alvarez-Venegas et al., 2007; March-Díaz et al., 2008; van den Burg and Takken, 2009; Jaskiewicz et al., 2011). Importantly, these epigenetic marks can allow a primed state to be transferred to the next generation (see the Transgenerational Effects section).

Priming is also common in primitive taxa, such as bacteria and biofilms, where it is often described as tolerance. Priming in response to pH (Foster and Hall, 1991; Shah, 2000), temperature (Boutibonnes et al., 1993; Walker et al., 1999; Desmond et al., 2004), salinity (Vargas et al., 2008), oxygen stress (Ahn et al., 2001), hydrostatic pressure (Ananta and Knorr, 2003), and several other sub-lethal stressors have been shown to increase tolerance to a range of different stressors (Hartke et al., 1994; Duwat et al., 2000; De Angelis and Gobbetti, 2004).

### Hormesis

Hormesis is an adaptive stress response identifiable by a biphasic nonlinear dose–response curve that exhibits a stimulatory (positive) effect at low stress levels and an inhibitory (negative) effect at higher stress levels (Calabrese and Baldwin, 1997). It is used to describe a biological response to stress, but can also occur in response to other stimuli that are not inherently harmful, such as nutritional signals. A hormetic dose response, shown in Figure 5D, is represented by a J- or U-shaped curve depending on whether the y-axis represents a parameter of normal biological function or dysfunction (Calabrese and Baldwin, 2001). Hormesis was coined in 1943 by Southam and Ehrlich (1943) to describe the enhanced metabolism of fungi exposed to Red Cedar extract, however, the stimulatory effects of low doses of toxins have been acknowledged since the 1880s. Although widely accepted in plant and microbial systems, it took longer for hormetic effects to be recognized in the field of mammalian toxicology. This was largely due to a perceived association with homeopathy but also to traditional medicine’s adoption of threshold dose–response models, which preclude the identification of responses in the low-dose zone (Calabrese, 2014). Today, hormesis has been documented in a diversity of taxa, from bacteria to humans, and documented for thousands of different compounds and physiological endpoints (Calabrese et al., 1999, 2008; Calabrese and Baldwin, 2003; Calabrese and Mattson, 2017). As such, it is now considered to be a fundamental biological response. Although hormesis has not been explicitly associated with anticipation or described as an anticipatory process (to my knowledge), it deserves mention here as it meets many of the criteria. Ultimately, more data are needed to make this determination, but a discussion of the relevant characteristics of hormesis and their relationship to the aforementioned criteria for anticipatory processes is discussed below.

Calabrese et al. (2007) describes three different types of hormesis: hormesis, which refers to the basic phenomena described above; conditioning hormesis, where prior exposure to a low-level stress reduces the effects of a subsequent higher exposure; and postexposure conditioning hormesis, where the negative effects of exposure to a high-level stressor is reduced by the subsequent exposure to a low-level stressor. As conditioning hormesis and postexposure conditioning hormesis relate more strongly to priming, this section will focus on the basic form of hormesis, i.e., the low-dose stress response in a naïve organism or cell. Hormesis is characterized as a type of general overcompensation to a homeostatic disturbance. The stimulatory effects observed at low stress exposure are presumed to stem from the unintended benefits of over-activated defense pathways in the absence of higher stress levels. Hormesis, for instance, can result in increased survival due to the upregulation of general defense pathways that inadvertently protect the organism from non-target sources of morbidity/mortality, as seen in dietary restriction. Even though, the stimulatory effects of hormesis are quite moderate, exhibiting a 30–60% change relative to control groups, it is difficult to derive an evolutionarily sound explanation for this sort of response, given that an over-reaction is an inherently inefficient response. One would expect natural selection to optimize stress responses so that they are adequate for mitigation but not excessive. Then again, one would not expect selection against any process that increases fitness. However, if we view hormesis as an anticipatory process, things begin to make more sense.

Just as biological processes can evolve to respond to temporal patterns in qualitative environmental signals, as in AAR, it is conceivable that they can also evolve to respond to temporal patterns in the quantitative aspects of environmental signals. Probabilistic relationships can exist between different events and also between different degrees of the same event. For instance, exposure to a specific amount of toxin could either indicate that a higher or lower dose is forthcoming. The relative probability of a higher or lower future dose depends on the shape of dose-time curve for the stressor in the environment and the position of the present dose along this trajectory. Like most environmental factors, the quantitative aspect of different stressors can vary through time in predictable ways, and this information can be integrated into stress response pathways and used to modulate responses in an anticipatory manner. In this way, the over-response observed at low doses of stress can be viewed as an attempt to mitigate the damage caused by higher future doses; but, interestingly, the benefits of hormesis do not derive from achieving this goal of enhanced preparedness. They instead originate from the preparation itself. Hormesis describes a situation where the benefits of an anticipatory response are evident in both the presence and absence of a future stressor. This makes hormesis a very unique type of response, as its utility appears to be dependent on the inaccurate prediction of a future state; in other words, on mounting a stress response for a stressor that never comes. While this may sound quite controversial, one could easily imagine a scenario where a stress response could prove beneficial under control conditions, particularly if it was only induced under very specific circumstances. Additionally, evolutionary theory does not predict strong selection against any phenotype that proves to be beneficial, even if it is the result of an erroneous prediction. A possible explanation for the maintenance hormesis may lie in the generality of the response. While many stress responses are very specific, hormesis appears to elicit a systemic generalized response that upregulates a suite of defensive pathways regardless of the causal stressor, similar to that of the universal cellular response (Nasonov, 1962; Matveev, 2005, 2010; Agutter, 2007). The up-regulation of specific stress-response pathways during control conditions would be of little benefit in the absence of that specific stress, but a more generalized response, like that observed in hormesis, could be beneficial, particularly since organisms must routinely contend with multiple stressors simultaneously. For instance, the activation detoxification pathways would be a waste of resources in the absence toxins because detoxification enzymes serve no other purpose than to aid in the excretion of toxic compounds, but the upregulation of processes protecting against oxidative stress, which is a natural byproduct of energy metabolism, would likely have positive effects under normal conditions.

Whatever the exact mechanism, it is important to note, as Forbes (2000) points out, that hormesis is not a phenomenon that acts directly on fitness, but instead is a homeostatic/allostatic mechanism that aims to maintain fitness across stressful conditions. As a result, the stimulatory effects of hormesis are likely subject to physiological trade-offs with other traits. An excellent example of this trade-off can be seen in dietary restriction (DR), a phenomenon where an increase in maximal and median lifespan is observed for organisms fed slightly deficient but not malnourishing diets (McCay et al., 1935; Piper et al., 2011). Dietary restriction has been documented across diverse taxa, from yeast to mammals (Mair and Dillin, 2008; Fontana et al., 2010), and represents a hormetic response where changes in diet, either a reduction in calories or specific macronutrients depending on the model organism, cause a systemic upregulation of cellular defense pathways that reduce a variety of age-related pathologies and lead to increased longevity (Mair et al., 2005; Masoro, 2007; Gems and Partridge, 2008; Simpson and Raubenheimer, 2009). It is a completely reversible response and, in most organisms, is associated with a concomitant reduction in reproductive output, indicating a trade-off between survival and reproduction (Lee et al., 2008; Moatt et al., 2016). The overriding evolutionary hypothesis for DR is that nutritional signals can act as indicators of environmental suitability, and when these signals change in a specific way, they indicate the presence of stressful conditions and elicit a biological response that prioritizes defense rather than reproduction. In other words, DR is biological program to “ride out the storm.” Several different nutrient-sensing pathways have been implicated in this response, including the insulin-like signaling pathway, the target or rapamycin pathway, and the AMP-kinase signaling pathways, with many downstream effects mediated by FOXO transcription factors (Fontana et al., 2010; Solon-Biet et al., 2015). Despite the many advancements in understanding DR, the mechanisms coordinating various defense pathways remain unknown.

### Transgenerational Effects

Lastly, I want to discuss how transgenerational effects may serve as a unique type of anticipatory processes; one whose purpose is to benefit the next generation. Like hormesis, transgenerational effects have not been described as anticipatory processes, but do meet many criteria. Up to this point, we have only discussed anticipation as it relates to individual organisms, particularly processes that maintain or increase an individual’s survival and/or performance across variable environments. Anticipation, however, can also be used to better prepare offspring for future events, *via* maternal/paternal effects and epigenetic marks (Angers et al., 2010; Bollati and Baccarelli, 2020; Leung et al., 2020). Transgenerational effects broadly refer to offspring phenotypes that are not dependent on changes in DNA sequence, and can occur when the conditions experienced by the parent either directly affect the cells that will become future offspring (maternal/paternal effects), or cause changes to epigenetic markers that can persist for multiple generations (epigenetic effects).

Parental effects refer to heritable phenotypes that are set in offspring before birth, either through the persistence of expressional states initiated in pre-zygotic cells (primordial germs cells or gametes), or through the passage of cytoplasmic signaling molecules, transcription factors, or RNAs from either parent during fertilization (Badyaev and Uller, 2009). Messenger RNAs, often preserved by polyadenylation (Winata et al., 2018), can be passed from the parent to the offspring to jumpstart gene expression (Ménézo, 2006; Stoeckius et al., 2014; Binder et al., 2015), in addition to various kinds of small RNAs (miRNA, siRNA, and piRNA). Other cytoplasmic factors can also impact gene expression in the offspring through direct effects on transcription or by mediating changes to gene methylation or chromatin structure (Kappeler and Meaney, 2010; Moreno-Romero et al., 2017; Tikhodeyev, 2018). Figure 5E shows a hypothetical example in plants where pest pressure experienced by the parent can cause an upregulation of resistance genes in their offspring *via* transgenerational effects.

Epigenetic effects are defined here as those mediated by alterations to epigenetic marks. There are many different types of epigenetic effects, however, not all of them are transgenerational or likely serve anticipatory functions. For example, paramutation, genomic imprinting, Polycomb silencing, and position effect variegation are epigenetic phenomena that show no specific relationship to environmental factors but instead are either randomly determined or operate only during development and differentiation (Martin and Zhang, 2007; Golbabapour et al., 2013). Other epigenetic marks, such as gene methylation and histone modifications, can carry information about past expressional states and persist across both mitosis and meiosis. If these marks are maintained in offspring or new cells, they can direct gene expression in adaptive ways. Because these marks serve as a sort of memory of expressional states, they are better candidates for carrying out anticipatory functions (Jaenisch and Bird, 2003; Angers et al., 2010; Bollati and Baccarelli, 2020; Leung and Gaudin, 2020). Gene methylation and histone modifications are the two most well-studied epigenetic marks, and while the causal relationship between gene expression and alterations to these marks are still ambiguous, the correlative patterns between them are reasonably consistent (Holliday, 1990, 2006; Karlić et al., 2010; Deans and Maggert, 2015). It is important to note that the distinction between parental and epigenetic effects hinges on the relationship between the past expressional state and how long it can be transmitted or “remembered.” When a parent is exposed to a particular environment, so too are their germ cells, hence it is impossible to separate the direct effects of gametic or embryonic exposure from the transfer of cytoplasmic compounds during fertilization. In mammals, exposure of an adult male to a particular environment also leads to the exposure of the F0 sperm cells within him. If a pregnant female is exposed to a particular environment, her eggs (F0), embryo (F1), and the primordial germ cells within the embryo (F2) are simultaneously exposed (up to three generations; Heard and Martienssen, 2014; Deans and Maggert, 2015). Parental effects, as defined here, are limited to the extent of this exposure, while epigenetic effects can persist in beyond three generations.

Gene methylation refers to the attachment of a methyl group to specific DNA bases. Gene methylation and demethylation are reversible covalent modifications carried out by methyltransferases. Typically, gene methylation results in the silencing of gene expression *via* the attraction of repressors, the inhibition of transcription factors, and/or alterations made to chromatin structure (Gibney and Nolan, 2010; Moore et al., 2013). The exact mechanisms that mediate methylation in response to environmental changes is not known, though *de novo* methylation in the parent and the selective maintenance of methylation marks during reprogramming are likely key factors (Angers et al., 2010; Bollati and Baccarelli, 2020). Much more research on the relationship between environmental factors and gene methylation is needed to better establish the role it may play in anticipatory processes, particularly as it relates to causal mechanisms.

Expressional memory can also be maintained and passed on through histone modifications that impact chromatin structure. Structure has important implications for gene expression, as transcription can only occur when the chromatin structure is loose enough to allow transcription machinery to interact with the DNA. Modifications on histone tails ultimately control chromatin structure, thus potentially impacting gene expression. Similar to gene methylation, these modifications may be maintained across meiosis and/or mitosis to impact expression in progeny cells and/or offspring (Holeski et al., 2012; Klosin et al., 2017); however, the connection between environmental variables and heritable histone modifications is ambiguous in many taxa, including mammals (Blake and Watson, 2016; Miska and Ferguson-Smith, 2016; van Otterdijk and MIchels, 2016).

Although much is still unknown about how and if environmental factors impact parental effects and epigenetic markers, transgenerational effects represent a potential anticipatory mechanism for information about parental environment to be stored and passed on to offspring to better prepare them for similar conditions. Because these mechanisms can increase the efficiency with which offspring respond to their environment, they may represent a unique type of anticipatory process. Parental effects and epigenetic markers are also not permanent and can be erased, or simply not maintained, if environmental conditions change, making this strategy flexible enough to respond to a dynamic environment.

### Summary

The anticipatory processes described in this section were selected because they meet at least some of the criteria outlined in the first section, however, their categorization is still largely conceptual. This is also far from an exhaustive list, and there is considerable overlap between many of these processes. For instance, plant eavesdropping often leads to priming in plants (Kessler et al., 2006; Morrell and Kessler, 2014). There are also many similarities between hormesis and priming (particularly priming and conditioning hormesis), with the main differences relating to the timeline and generality of the response. One must wonder if these processes represent uniquely evolved responses, different stages of a common evolutionary trajectory, or a shared underlying mechanism. Given that underlying mechanisms for most of these processes are not well understood, it is also possible that some of these responses are redundant and represent the same mechanism. A good example of this ambiguity can be seen in the phenomenon of DR, as it has been suggested that DR represents a hormetic response but also bears the hallmarks of AAR, with defensive pathways responding to nutritional cues as a general indicator of future stress. Much more work will be needed to understand the relationship between these responses, which for too long have been study in isolation. Perhaps the greatest obstacle will be characterizing the mechanisms at work; a topic which is discussed in the following section.

## Mechanisms of Anticipation

A preeminent goal in systems biology is to understand how living systems remain robust to environmental perturbations. We have already discussed the adaptive role that anticipatory processes play in homeostasis/allostasis but have yet to discuss the specific mechanisms that mediate these responses. While information on anticipatory mechanisms is still quite limited, control systems theory, network analyses, and dynamical systems modeling have been broadly employed to understand different types of biological mechanisms. In particular, control system theory has been useful for understanding homeostatic regulation, through the acknowledgement that optimization in both living and manmade systems operates to reduce error. Given that we have already defined anticipatory systems in terms of error, we can use these same principles to discuss potential anticipatory mechanisms. It has also been shown that certain network topologies and designs can be reliably associated with specific system properties, i.e., signal sorting, filtering, delays, synchronization (Mangan et al., 2003; Mangan and Alon, 2003). While the role that these properties and other types of system dynamics play in anticipatory mechanisms is still unknown, theoretical work has shown that anticipation can be produced under certain designs. For instance, master–slave systems have been shown to produce anticipatory synchronization under certain parameters. The following sections summarize what is currently known and discusses the potential link between existing designs and biological anticipatory mechanisms.

### Feed-Forward Control

The primary application of control systems theory in biology acts to understand how biological systems mitigate homeostatic error; particularly how they maintain function in the face of fluctuations (robustness), and how precisely and quickly they can reset back to an undisturbed state (adaptation). Generally speaking, there are two main types of control systems: feedback and feed-forward control. In a feedback control system, the output is quantitatively compared to the input to generate a measure of error. This error signal is then filtered out by a feedback controller that adjusts the input going back into the system. Feedback control represents a closed-loop design that mitigates error after it occurs, much in the same way that traditional models of homeostasis operate. Alternatively, feed-forward control systems are open designs with controllers that adjust for error before the input enters the system. In this setup, environmental error is predicted or predetermined and applied to the input before it enters the systems to avoid or reduce system error, similarly to the process described in allostasis. There is an obvious connection between feed-forward control and anticipatory processes, as both rely on predictions about future states to minimize error in adaptive ways.

There are a multitude of examples showing how feed-forward control is used in biological processes to deal with environmental fluctuations, particularly in cognitive taxa (Fajas et al., 1998; Yeo et al., 2016) but fewer examples available for non-cognitive organisms (Franks et al., 1997; Shudo et al., 2003; El-Samad, 2010). Control systems were discussed by Rosen (1985a) and later Poli (2009, 2010) in reference to anticipation, yet a strong empirical connection between feed-forward control and biological anticipation remains to be substantiated, although, examples do exist. Shudo et al. (2003) and El-Samad (2010) describe how the heat-shock response in *E. coli* uses feed-forward control to anticipate the need for heat-shock protein (HSP) production. In the *E. coli*, the transcription of HSP genes is regulated by the σ32 protein. The mRNA of the rpoH gene, which encodes the σ32 protein, is bound to itself at low temperatures but unfolds at high temperatures as the secondary structure relaxes. This allows RNA polymerase to bind to the promoter and transcription/translation of HSPs to take place. The translation of the σ32 protein happens before any heat-induced damage to cellular proteins occurs. In this way, the σ32 protein serves as a temperature sensor and an anticipatory stimulus, and because it modulates the production of HSPs based on an anticipated future need, it represents a feed-forward control system. In addition to HSPs, σ32 also upregulates the expression of the chaperones DnaK and DnaJ, which control σ32 activity through a sequestration feedback loop by binding σ32 at low concentrations of misfolded proteins. This binding not only halts the expression of HSPs but leads to the degradation of σ32 by Lon and FtsH proteases.

Another example of feed-forward control in *E. coli* is the anticipatory expression of alkalinization defense genes by trimethylamine N-oxide (TMAO). Bordi et al. (2003) describe how the TorS/TorR regulatory system modulates the activity of the anaerobic TMAO reductase respiratory system, which utilizes methylamines as substrates. TMAO is reduced to TMA by TMAO reductase as part of this process and TMA acts as weak base that increases intracellular pH. They found that the presence of TMAO upregulated the expression of TMAO reductase respiratory machinery but also defense genes related to extreme pH. Again, in this system TMAO acts as an anticipatory stimulus and is used as a predictor of impending pH stress to modulate the expression alkalization defense genes through a feed-forward control design.

I want to note that feed-forward control and feed-forward motifs, which are used to describe regulatory network topologies in biochemical circuits, are not the same thing. In the literature, these concepts are unfortunately easily conflated. Feed-forward loops (FFLs) are common regulatory network motifs found largely in microbial transcriptional networks (Mangan et al., 2003; Mangan and Alon, 2003; Milo et al., 2004; Alon, 2007; Albanesi et al., 2013; Mank et al., 2013; Westerhoff et al., 2014). They describe three-node networks where one transcription factor directly regulates a target gene and also indirectly regulates the same gene through a secondary transcription factor. There are eight possible configurations of FFLs, which depend on whether the interactions between the nodes are positive or negative and whether they display AND or OR logic (Mangan and Alon, 2003; Milo et al., 2004; Alon, 2007). While incoherent FFL designs are associated with accelerated response times and have been implicated in perfect adaptation (Westerhoff et al., 2014), these network motifs do not possess an inherent connection to feed-forward control systems or anticipation and have only been implicated in reactive processes. Although FFLs are likely present in both anticipatory and reactive regulatory circuits, and thus do play a role in relating stimuli with their respective responses, there is currently no evidence for their role in anticipatory functions. In fact, no specific network motif has been implicated in anticipatory mechanisms. As discussed in Lipshtat et al. (2008), in order to understand the function of different network motifs they must be placed in the context of other motifs because the overall structure and organization ultimately shapes input–output relationships. This notion is also imperative for understanding anticipatory mechanisms, as the entire system has to be accounted for, from the foremost stimulus to the endpoint of the response, in order to characterize a process as reactive or anticipatory. This is rarely done when studying fine-scale mechanisms, which highlights the importance of understanding biological phenomena on multiple levels; from the evolutionary scale to the biochemical scale. That being said, determining whether specific network motifs or circuit designs are more prevalent in anticipatory processes will be useful for further elucidating the mechanistic details of these processes.

### Anticipatory Synchronization

The production of anticipatory synchronized oscillations is an example of one way that system dynamics can mediate anticipation. Anticipatory synchronization (AS) can be achieved by coupling two identical dynamic systems in a master–slave configuration (Voss, 2000; Matias et al., 2011). By subjecting the slave to a certain degree of delayed negative feedback, a stable state emerges where the state of the slave at time t is in the same state that the master will be in at a future time. In this way, the slave anticipates the future state of the master through AS. Anticipatory synchronization has been demonstrated in diverse models, including discrete and continuous systems, as well as noisy, chaotic, excitable, and quasi-periodic dynamic systems (Voss, 2000; Masoller, 2001; Hernández-García et al., 2002; Ciszak et al., 2003; Masoller and Zanette, 2003; Calvo et al., 2004), producing both short- and long-term anticipation times (Pyragas and Pyragienė, 2008). Anticipatory synchronization has been experimentally demonstrated using electronic circuits (Voss, 2002; Ciszak et al., 2003, 2009) and lasers (Ahlers et al., 1998; Heil et al., 2001; Sivaprakasam et al., 2001; Buldú et al., 2002; Liu et al., 2002). Matias et al. (2011) showed that AS could be produced in a 3-neuron Hodgkin–Huxley-type model under certain parameters, and Ciszak et al. (2009) even showed how AS could be used to suppress spikes in a perturbed FitzHugh-Nagumo neuronal system by incorporating AS into a feed-forward design. Although oscillations are common in living things, including circadian rhythms, cytoplasmic oscillators such as glycolytic cycles, cell cycles, neuronal membrane oscillators, and transcriptional oscillations, it is not yet established whether the AS found in physical systems are also present in biological systems.

## Discussion

An unavoidable disadvantage of working within any paradigm, is that we are only able to see those things that fit inside of it. It is an age-old epistemological problem. Rosen’s view was that modern science, being largely shaped by the fields of physics and mathematics, leads us to view biology in a purely “reactive” way, and one that has marginalized the role of anticipation in biological systems. Whether you agree with Rosen’s supposition or not, the topics of anticipation have been curiously limited in the greater biological sciences, despite a history of evidence that spans over 100 years. One could invoke the law of parsimony to justify or explain this, as anticipatory designs do seem less intuitive and perhaps more complex than reactive ones; but are they really? Are feed-forward systems more complex than feedback systems? One could argue that there is no inherent difference in complexity, simply a difference in organization. In any case, if a primary goal of biology is to understand the diversity of biological processes and functions, the argument is largely moot, for anticipatory processes most definitely exist and therefore must have a place in the field of biology.

It is bombastic to suggest that accounting for anticipatory processes requires a reworking of fundamental biological principles; however, anticipatory processes do have important implications for how we view and understand the mechanistic features of biological processes and their evolution. In this regard, the existence of anticipatory processes impacts both the proximate and ultimate factors surrounding biological function. Also, because the differences between reactive and anticipatory processes occur at a causal level, they have the ability to impact many successive qualities. Making this change, however, does not require the destruction of any standing principles, simply the addition of new considerations, especially regarding topics related to the regulatory properties of these processes and contexts in which they develop. Much like the acknowledgement of epigenetic mechanisms has not erased Mendelian principles but simply expanded the field in a way that has helped explain many aspects of gene function that would otherwise remain inexplicable, incorporating the concept of anticipatory mechanisms into our current understanding of biological function need not defy Newtonian principles, nor does it require additional parts. It simply requires the open-mindedness to consider the reorganization of existing parts.

Much more work is needed to further understand anticipatory processes, including their evolution, role in biological function, and mechanisms. Many of the concepts put forth in this article are admittedly hypothetical; however, it is my hope that they will help inform and direct future research on these aspects of biological anticipation. In particularly, further documenting the existence of other types of anticipatory processes and the prevalence of these mechanisms should be of chief importance. For instance, identifying the relative proportion of biological processes that are regulated by anticipatory vs. reactive mechanisms in specific model species will be key to substantiating these as fundamental biological mechanisms. There is a great opportunity for the development of novel computational methods to play a primary role in this pursuit, particularly as it relates to understanding the regulatory structure and dynamics of anticipatory processes. Comparative approaches that focus on highly conserved anticipatory mechanisms, such as dietary restriction and circadian-regulated processes, provide an opportunity to learn more about the evolution of these mechanisms and the constraints to their development. Artificial selection studies using microbial models will also be useful for understanding the environmental conditions that are conducive to the evolution of anticipatory processes and how they are maintained. Understanding the evolution of anticipatory processes and their mechanisms is key to broadening our understanding of how organisms deal with environmental change, and this is particularly important given the impending challenges of global climate change and urbanization. In applied fields, anticipatory mechanisms may 1 day provide new constructs for the development of novel biotechnology and bioengineering solutions. Ultimately, it is only through the integration of an anticipatory perspective with the current reactive paradigm that we can effectively expand our understanding of complex biological processes to produce functional knowledge.

## Author Contributions

CD is responsible for the writing of this article, the creation of all figures, and the generation of the concepts and ideas provided.

## Funding

This work was done while supported on a Minnesota Department of Agriculture AGRI Crop Research Grant in Dr. William Hutchison’s lab at the University of Minnesota.

## Conflict of Interest

The author declares that the research was conducted in the absence of any commercial or financial relationships that could be construed as a potential conflict of interest.

## Publisher’s Note

All claims expressed in this article are solely those of the authors and do not necessarily represent those of their affiliated organizations, or those of the publisher, the editors and the reviewers. Any product that may be evaluated in this article, or claim that may be made by its manufacturer, is not guaranteed or endorsed by the publisher.
